# The impact of female obesity on the outcome of fertility treatment

**DOI:** 10.4103/0974-1208.69332

**Published:** 2010

**Authors:** Shilpi Pandey, Suruchi Pandey, Abha Maheshwari, Siladitya Bhattacharya

**Affiliations:** Assisted Reproduction Unit, University of Aberdeen, Aberdeen Maternity Hospital, Aberdeen, United Kingdom; 1 Department of Obstetrics and Gynaecology, Stoke Mandeville Hospital, Aylesbury, United Kingdom

**Keywords:** Assisted reproduction, fertility treatment, obesity

## Abstract

The rising prevalence of obesity has had a profound impact on female reproductive health. Increased body mass index (BMI) is associated with ovulatory subfertility and anovulatory infertility. Overweight and obese women have poorer outcomes following fertility treatment. They respond poorly to clomiphene induction of ovulation and require higher doses of gonadotrophins for ovulation induction and superovulation. Ovarian stimulation for assisted reproduction produces fewer follicles resulting in the harvest of fewer oocytes. Fertilization rates are poorer and the embryo quality is impaired in younger women who are obese. Pregnancy rate in some studies is lower and there is an increased risk of early pregnancy loss. Weight loss regularizes menstrual cycles and increases the chance of spontaneous ovulation and conception in anovulatory overweight and obese women. Gradual sustained weight loss is beneficial whereas crash dieting is detrimental.

## INTRODUCTION

Rising obesity rates present a global public health challenge. Approximately 1.6 billion adults worldwide were overweight (BMI 25-30 kg/m^2^) and at least 400 million were obese (BMI >30 kg/m^2^) in 2005. These figures are expected to rise to 2.3 billion and 700 million, respectively, by 2015.[1] In the West, 56% of women in England[2] and 61% women in the United States are believed to be either overweight or obese.[3] The prevalence of obesity is relatively low in Asian countries with 4% of Chinese and 0.5% of Indian women noted to be obese.[4] Nevertheless, in the last 20 years, the obesity rates have tripled in the developing world and 10% of all children across the world are overweight or obese.[5]

The World Health Organization has defined obesity as body mass index (BMI) ≥ 30 kg/m^2^ [Table 1]. Obesity is associated with cardiovascular disease, diabetes, osteoarthritis and malignancies such as colon and endometrial cancer.[1] It is increasingly being recognized that this current obesity epidemic has also contributed to fertility problems. To explore the impact of obesity on fertility and outcome of fertility treatment, a literature search was performed on Medline covering a period from 1950 to July 2010 and EMBASE from 1980 to July 2010, using the search terms ‘obesity’, ‘female infertility’, ‘ovulation induction’, ’super ovulation IUI’, ‘donor IUI’, ‘assisted reproduction’, ‘IVF’, ‘ICSI’, ‘pregnancy rate’, ‘miscarriage rate’ and ‘live birth’.

**Table 1:** WHO classification of obesity

	BMI
Under weight	<18.5
Normal weight	18.5-24.9
Over weight	25.0-29.9
Obesity class 1	30.0-34.9
Obesity class 2	35.0-39.9
Obesity class 3	40+

## IMPACT OF OBESITY ON FERTILITY

Obesity in women has been shown to increase time to conception.[6–8] The relative risk of anovulatory infertility is 2.7 (95% CI, 2.0-3.7) in women with BMI≥ 32 kg/m^2^at age 18,[9] while in ovulatory but subfertile woman the chance of spontaneous conception decreases by 5%[10] for each unit increase in the BMI.

The mechanisms by which obesity causes or exacerbates subfertility are manifold. High BMI is associated with an increase in serum and follicular fluid leptin concentration[11] and decrease in serum adiponectin levels.[12] Leptin acting through the receptors on the theca and granulosa cells inhibits ovarian steroidogenesis.[13–15] Lower adiponectin levels are associated with increased circulating insulin[12] which can cause hyperandrogenaemia partly by inhibiting the hepatic SHBG (sex hormone binding globulin) production.[11] In addition, insulin acting via IGF1(insulin like growth factor 1) enhances LH mediated steroidogenesis in the theca cell system of the ovary and thus increases ovarian androgens.[16] Hyperandrogenaemia results in granulosa cell apoptosis, while peripheral conversion of androgens to estrogen in adipose tissue inhibits gonadotrophin secretion.[11]

Obesity is also associated with polycystic ovary syndrome (PCOS) which is a heterogeneous condition characterized by oligo or anovulation, hyperandrogenism, menstrual irregularities and subfertilty.[17,18] Obesity which occurs in 30-75% of women with PCOS[19] increases the magnitude of hormonal and metabolic dysfunction in these women.[18]

## IMPACT OF OBESITY ON FERTILITY TREATMENT

To assess the impact of obesity on fertility treatment, we need to assess the impact of obesity on ovulation induction for anovulatory women, superovulation IUI for ovulatory subfertile women and donor insemination for severe male factor.

### Ovulation induction

The evidence for the impact of obesity on ovulation induction treatment is conflicting. Obese women tend to respond poorly to ovulation induction using clomiphene citrate[20] and pregnancy rates are lower[21] in those who do.

A systematic review of 13 studies suggests that obesity and insulin resistance are predictors of suboptimal outcomes following ovulation induction using gonadotrophins. Women with high BMI need higher total doses of FSH to achieve ovulation [weighted mean difference 771 IU (95% CI, 700–842)]. These women also face a higher risk of cycle cancellation [OR 1.86 (95% CI: 1.13–3.06)] and are less likely to ovulate [OR 0.44 (95% CI: 0.31–0.61)].[22] However, in a multicenter randomized controlled trial involving 335 women with WHO type II anovulatory infertility, although those with higher BMI took longer to ovulate despite increased doses of gonadotrophins, rates of ovulation and clinical pregnancy were comparable to those in normal BMI women.[23]

### Superovulation IUI

In a study exploring the impact of obesity on outcomes of superovulation IUI treatment, there was no statistically significant difference in adjusted cycle fecundity and mean number of large follicles, following superovulation IUI among normal weight, overweight and obese women.[24] Compared to women with BMI ≥18.5<25 kg/m^2^, the odds ratio of cycle fecundity for women with BMI ≥25<30kg/m^2^ was 0.70 (95%CI 0.35-1.40) and those for women with BMI>30 kg/m^2^ was 0.87 (95% CI. 0.35-2.18).[24] Obese women, however, had lower peak estradiol levels and required higher doses and longer duration of gonadotrophins injections compared to overweight and normal weight women.[24]

### Donor insemination

Donor insemination is the treatment option for couples with severe male factor (azoospermia). In a study involving 1144 women undergoing donor insemination, the authors concluded that pregnancy rate was 42% among women with BMI 20-24 kg/m^2^, 33% for women with BMI 25-27 kg/m^2^, and 21% for women with BMI 28-36 kg/m^2^.[25] Thus, the pregnancy rates progressively decrease with increasing BMI. Another study concluded that the cumulative conception rate after 12 cycles was 48% for normal weight (BMI 20-25) and overweight women (BMI 25-30), but only 18% for obese women BMI>30 kg/m^2^.[26] They further concluded that the waist hip ratio was a better predictor of outcome than BMI.[26] Each 0.1 unit increase in waist hip ratio resulted in 30% decline in probability of conception per cycle (hazard ratio 0.706; 95% CI 0.562 to 0.887).[26]

## IMPACT OF OBESITY ON ASSISTED REPRODUCTION

In the following section, we would like to highlight the impact of obesity on each individual component of in-vitro fertilization.

### Ovarian stimulation

Gonadotrophin requirements are higher in overweight and obese women who have an increased incidence of poor ovarian response. A systematic review of IVF outcomes among overweight and obese women demonstrated that the dose of gonadotrophins required was higher in women with BMI of>25 kg/m^2^ [weighted mean difference (WMD) 210.08, 95% CI: 149.12, 271.05] in comparison with those with BMI of <25 kg/m^2^. Gonadotrophin requirements were higher (WMD 361.94, 95% CI: 156.47, 567.40) in obese women (BMI>30 kg/m^2^) when compared to non obese women.[27] A large cohort study published after the systematic review[28] showed that overweight women required more ampoules of gonadotrophin (*P*<0.002), had lower peak oestradiol levels (*P*<0.001) and faced an increased risk of cycle cancellation due to poor follicular development (*P*<0.018)[28] [Table 2].

**Table 2:** Impact of obesity on ART

Ovarian stimulation	
Gonadotrophin requirement	Increased
Response to stimulation	Poorer
Oocyte number	Reduced
Oocyte quality	Unchanged
Fertilization	Decreased
Embryo quality	Poorer in some studies
Cycle cancellation	Insufficient evidence
OHSS	Insufficient evidence
Pregnancy rate	Reduced in some studies
Miscarriage rate	Increased
Livebirth	Insufficient evidence

OHSS - Ovarian hyperstimulation syndrome

### Oocyte recovery

The procedure of oocyte recovery is more challenging in women with high BMI. Obese patients have difficult venous access.[29] General anesthesia can be more hazardous while response to conscious sedation may be erratic with a higher risk of hypoxaemia.[30]

### Oocyte number and oocyte quality

A large cohort study has shown that in comparison with women of normal weight, overweight women (BMI>25<30 kg/m^2^) have significantly fewer oocytes retrieved (12.98± 6.91 *vs*. 14.49±7.96, *P*<0.001).[31]

These findings were supported by a systematic review where the weighted mean difference (WMD) of the number of oocytes recovered in women with BMI >25 kg/m^2^ was 0.58 (95% CI: 0.22, 0.94) in comparison with women with BMI <25 kg/m^2^.[27] Another study suggested that oocyte quality (demonstrated by number of oocytes considered suitable for injection or the number that fertilized) was unaffected by BMI.[32]

### Oocyte fertilization and embryo quality

Oocyte fertilization rates have been shown to be lower in morbidly obese women (59% *vs*. 69%; *P*<0.03)[33] [Table 2]. A large cohort study has shown that in comparison with women of normal weight, overweight women (BMI>25<30 kg/m^2^) have lower fertilization rates (60.8±23.3 *vs*. 61.1±23.0, *P*<0.001), fewer cleaved embryos (7.55±4.86 *vs*. 8.67 ±5.90, *P*<0.001), fewer high-grade embryos (4.65±3.96 *vs*. 5.59±4.81, *P*<0.001) and fewer cryo preserved embryos (4.44±4.55 *vs*. 5.49±5.55, *P*<0.001).[31] Another study demonstrated that embryo quality (reflected by embryo grade, embryo utilization and cryopreservation) in women under 35 years of age was poorer in those who were obese.[32] A large retrospective study on 6500 IVF/ICSI cycles, however, concluded that the embryo quality was not impaired in overweight and obese women.[34] This can be explained by the fact that the average age of women in this study was between 34 and 35 years and does not appear to be in conflict with the previous findings.

### Cycle cancellation

Several studies have looked at the cycle cancellations in overweight and obese women. The systematic review by Maheshwari *et al*,[27] suggests that the odds of cycle cancellation in women with BMI of >25 kg/m^2^ was 1.83 (95%CI: 1.36, 2.45) as compared to women with BMI <25 kg/m^2^. However, their pooled data displayed evidence of significant statistical heterogeneity (*P*<0.05) and for women with BMI >30 kg/m^2^ the results were unable to confirm an increase in the risk of cycle cancellation. A recent study, however, demonstrated that the prevalence of poor responders was significantly higher among obese than non-obese women (28.2% vs 16.9%, *P*<0.04)[35] [Table 2].

### Ovarian hyperstimulation syndrome

Ovarian hyperstimulation syndrome (OHSS) is an avoidable complication of ovarian stimulation. A systematic review has not shown a statistically significant increase in the risk of OHSS among overweight and obese women. The authors of this review highlighted the fact that their conclusion was based on relatively small numbers of cases due to inconsistencies of reporting OHSS as an outcome.[27]

### Implantation, pregnancy and live birth rates

A systematic review concluded that, in comparison with women of BMI >25, those with BMI 20–25 kg/m^2^ had a higher chance of achieving pregnancy [combined odds ratio = 1.40 (95% CI: 1.22, 1.60)]. The combined odds of pregnancy were 1.47 (95% CI: 1.20, 1.80) for a woman with a BMI 20-30 kg/m^2^ as compared to women with a BMI of >30 kg/m^2^.[27] This meta-analysis of aggregated observational data was unable to adjust for confounders such as age, duration of infertility and previous pregnancy. It was also unable to come to any firm conclusions about the impact of obesity on IVF live birth rates due to insufficient evidence.[27] Some recent studies[28,31] have failed to confirm a direct association between high BMI and reduced live birth rates. Thus, we can conclude that while there is evidence linking obesity with poor implantation and pregnancy rates, more robust studies are needed to substantiate this [Table 2].

### Early pregnancy loss

There is an increased risk of miscarriage in overweight and obese women after spontaneous conception, ovulation induction,[36] IVF[37] and oocyte donation.[38]

A systematic review of literature showed that when compared with women of BMI<25, the odds of miscarriage in women with BMI of ≥ 25 kg/m^2^ were 1.33(95% CI: 1.06-1.638). The odds of miscarriage were 1.53(95% CI: 1.27-1.84)[27] in women with BMI≥ 30 kg/m^2^ when compared to women of BMI< 30 kg/m^2^. However, the authors of this meta-analysis were unable to adjust for confounders such as age and co-morbidities such as PCOS as individual patient data were not available to them.

Another recent meta-analysis of the available evidence suggested that there was a significant increase in the odds of miscarriage in women with a BMI of ≥ 25 kg/m^2^ (OR1.67; 95% CI, 1.25–2.25) following spontaneously conceived pregnancies as well as following ovulation induction (OR, 5.11; 95% CI, 1.76–14.83).[39] However, there was no evidence for increased miscarriage rates in high BMI women who underwent IVF/ ICSI (OR, 1.52, 95% CI, 0.88–2.61). These results were differing from those in the previously quoted systematic review due to differences in the nature of the included studies and the type of denominator used[39] to calculate the prevalence of miscarriage.

The reasons behind an increased risk of miscarriage amid overweight or obese women have been debated. It has been suggested that this is due to impaired folliculogenesis and poor oocyte quality in obese women. The proponents of this view cite a cohort study which concluded that uterine receptivity was unimpaired in women with increased BMI when hormonal support and embryo quality were standardized.[40] The alternative hypothesis is that endometrial receptivity is impaired in the overweight and obese women. A study involving 2656 ovum donation cycles with good quality embryos suggested that the ongoing pregnancy rate per cycle was poorer in the overweight and obese recipients than in the underweight and normal groups. Women under 25 kg/m^2^ had an ongoing pregnancy rate per cycle of 45.5%, compared with 38.3% for those with BMI> 25 kg/m^2^.[41] In another study based on 6500 IVF/ ICSI cycles, there was no difference in the quality of embryos, but implantation, pregnancy, and live birth rates were poorer in obese women.[33]

Most authors agree that there is an increased risk of miscarriage in overweight and obese women after spontaneous conception; however, any further increase in risk after IVF or ICSI is debatable. The reasons of an increased risk of miscarriage could possibly be the higher prevalence of PCOS among overweight and obese women.

Due to the adverse impact of BMI on treatment outcomes, there have been calls for restricting publically funded fertility treatment to women with high BMI. The threshold BMI used as a cut off value for restricting access to publically funded fertility treatment varies around the world. Across the UK, there is no uniformity of practice with cut-off values for access to IVF varying from 30 kg/m^2^ to 35 kg/m^2^.[42] The NICE fertility guideline in the United Kingdom suggests a BMI ≤ 29 is ideal.[43] British Fertility Society guidance suggests that fertility treatment should be deferred until BMI is less than 35 kg/m^2^, although in those younger than 37 years with normal FSH, weight reduction to BMI less than 30 kg/m^2^ is preferred.[44] A 5-10% weight loss has been recommended for overweight and obese women.

### Benefits of weight loss

Available data suggest that as little as 5%–10% weight loss can improve fertility outcomes.[45] Other studies have demonstrated that 5% weight loss results in improvement of endocrine parameters, such as decrease in free testosterone, lower fasting insulin levels and increased frequency of ovulation.[46] In addition, weight loss causes a significant reduction in central fat deposits (11%) and serum luteinizing hormone levels[47] with return of normal menstrual cycles in four out of five women.[48]

Weight loss can be achieved by lifestyle modification, dietary restriction, physical activity and pharmacotherapy with varied results. Life style modification programs (especially diet programs) have been shown to be associated with poor levels of compliance[49] and are not particularly suitable for women who want to conceive soon.[50] Dietary interventions are associated with increasing weight regain over time, although this can be minimized with continuing care.[51] Only 15% of the subjects can sustain weight loss successfully over time and there is a positive effect of group therapy, behaviour modification and active follow-up.[52] Although enhanced reproductive function may be induced by caloric deficit and relatively small weight loss, the maintenance of reduced weight may be critical for reduced complications during pregnancy and birth and for reduction of cardiovascular and diabetic morbidity and mortality.[49] Rapid weight loss achieved by crash diets or excessive exercise is detrimental to reproductive outcomes during fertility treatments. A study of very low calorie diet resulting in 8.8% weight loss in a six week period had to be suspended due to its impact on oocyte quality and fertilization rates.[53]

In a large randomized controlled trial pharmacological measures like metformin, at a dose of 850 mg twice daily, have not been shown to affect menstrual frequency, body weight or insulin sensitivity, despite a fall in total testosterone and waist circumference.[54] There have been small prospective studies on the use of Orlistat in obese PCOS showing a degree of effectiveness; however, there are no large randomized controlled trials in obese subfertile women.[55]

Decision making around postponement of fertility treatment to allow weight loss to take place needs to accommodate the effect of further increase in age in older women. A recent study demonstrated that the effect of BMI on IVF success was strongly influenced by age. With increasing age, the effect of BMI alone was attenuated such that, it became less influential than age, in women aged 36 years or older.[56]

## CONCLUSION

Obesity in women has impacts on fertility and fertility treatment. Increase in BMI reduces the chance of conception in ovulatory women and affects the outcome of ovulation induction treatment.

Obese women undergoing IVF require higher doses of gonadotrophins, respond poorly to ovarian stimulation and have fewer oocytes harvested. Obesity is associated with lower fertilization rates, poor quality embryos and higher miscarriage rates. Weight loss in these women improves their reproductive outcomes; however, in order for this to be effective it has to be gradual and sustained.
